# Outcomes of microwave ablation for hepatocellular carcinoma adjacent to large vessels: a propensity score analysis

**DOI:** 10.18632/oncotarget.15672

**Published:** 2017-02-24

**Authors:** Jian-ping Dou, Jie Yu, Xiao-huan Yang, Zhi-gang Cheng, Zhi-yu Han, Fang-yi Liu, Xiao-ling Yu, Ping Liang

**Affiliations:** ^1^ Department of Interventional Ultrasound, Chinese People's Liberation Army (PLA) General Hospital, Beijing, 100853, China

**Keywords:** hepatocellular carcinoma, microwave ablation, vessel, prognosis, propensity score matching

## Abstract

The aim of this study was to retrospectively compare the long-term efficacy of MW ablation as a curative therapy for hepatocellular carcinoma(HCC) adjacent to large vessels(≥3 mm) with that in safe location. Between 2010 and 2016, 406 patients diagnosed with early-stage HCC at Chinese PLA general hospital were enrolled. One-to-one matched pairs between the vessel group and the safe group were generated using propensity score matching. The associations of treatment strategy with overall survival and local tumor progression were determined by Cox regression. Before matching, 113 patients were classified into the vessel group and 293 patients were classified into the vessel group. The patients in the vessel group were more frequently classified as larger tumor size (P<0.05) and higher AFP level (P<0.05) than patients in the safe group. After propensity score matching, 113 pairs of well-matched HCC patients were selected from different treatment groups. No significant differences were found in local tumor progression, overall survival and complication rates for MW ablation as a first-line treatment for the early-stage HCC between two groups. In conclusion, MW ablation provides an effective and safe way to treat early-stage HCC adjacent to large vessels.

## INTRODUCTION

Minimally invasive thermal ablation of tumors has become common since the advent of modern imaging. With all those years’ development, thermal ablation has broadened its application area from the treatment of small, unresectable tumors or for patients who are poor surgical candidates to a curative therapy for patients with early-stage HCC [1].

It used to be believed that ablation for tumors adjacent to large vessels, liver surface, biliary tree, or near to bowel should be avoided [2] for the concern of major complications (SIR classifications C–E)[3] or the incomplete coagulation. For tumors adjacent to large vessels, three major issues should be focused. First, large vessels close to the tumor could take the heat away to flowing blood and prevent complete ablation, which is known as heat-sink effects. This effect could cause a higher local tumor progression (LTP) rate [4–6]. Second, the heat might cause damage to surrounding vessels, decrease the supple of liver and even cause liver failure [7–9]. Third, some studies reported that an increase in intratumoral pressure during ablation could cause dislodgement and spread of cancer cells to the remote part of the liver through surrounding vessels [10–11]. Microwave (MW) ablation seemed to be a better choice than radiofrequency (RF) ablation in treating HCC adjacent to large vessels, for its advantages of a lower susceptibility to heat-sink effects (blood-vessel-mediated cooling), as well as the ability to achieve larger tumor volumes in shorter time [12]. Related studies have been published in those years. Due to the small sample size, confounding bias or lax inclusion criteria, published studies were flawed to confirm the theoretical advantages of MW in ablating tumors adjacent to large vessels in clinical practice [13–15].

Propensity score matching (PSM) is an emerging statistical technique used in the statistical analysis of observational data. It could well reduce the bias due to confounding variables that could be found in an estimate of the treatment effect obtained from simply comparing outcomes among units that received the treatment versus those that did not [16]. Use of PSM analysis would help to make some reliable conclusions to the outcome of MW ablation for HCC adjacent to large vessels. To the best of our knowledge, no such studies have been published. In this study, we used PSM analysis to retrospectively compare the long-term results of percutaneous MW ablation as a curative therapy for HCC adjacent to large vessels with those in safe location.

## RESULTS

### Baseline characteristics

Over a 6-year period, 406 patients with 557 HCC lesions were included in this study. HCC was diagnosed according to pathologic findings in 294 patients and imaging findings in 112 patients. The median follow-up period was 29.3 months (range, 6.0–76 months). The baseline characteristics of all patients were shown in Table 1. Before matching, the vessel group has larger tumors than the safe group (2.76cm vs 2.47cm, respectively; p<0.05). The proportion of high AFP level (>200 ng/mL) was 16.0% in the safe group and 26.5% in the vessel group (P <0.05). Median follow-up time was 29.6 months (range, 6–76 months) in the vessel group and 29.2 months (range, 6–75 months) in the safe group, without a significant difference between the two groups (P>0.05). 226 patients were selected in the propensity score-matched cohort and 180 patients were excluded. After propensity score matching, patients in the two groups were not significantly different with regard to any baseline factors (Table 1).

**Table 1:** Baseline characteristics of study patients before and after propensity score analysis

Factor	Before Propensity Score Matching			After Propensity Score Matching		
	Safe group	Vessel group	P Value	Safe group	Vessel group	P Value
**Age(y)**	58.7	59.4	0.542	60.28	59.43	0.574
**NO. of men**	233	91	0.820	90	91	0.798
**Tumor size**	2.47	2.76	0.011	2.858	2.765	0.538
**AFP level >200 ng/mL**	47(293)	30(113)	0.016	22	30	0.206
**Origin of chronic liver disease**			0.905			0.961
** Hepatitis B virus**	240	90		87	90	
** Hepatitis C virus**	36	17		20	17	
** Other origin**	3	1		1	1	
** None**	14	5		5	5	
**Presence of liver cirrhosis**			0.785			0.472
** yes**	278	107		110	108	
** no**	15	5		3	5	
**Child-Pugh class**			0.762			>0.99
** Class A**	289	111		111	111	
** Class B**	4	2		2	2	
**NLR before ablation**	2.16	2.07	0.622	2.17	2.07	0.648
**ALT(U/L)**	35.39	35.06	0.907	34.05	35.07	0.745
**PLT (10^<sup>9</sup>^/L)**	109.02	110.01	0.908	118.3	106.01	0.000
**Follow-up time(mo)**	29.2	29.6	0.809	27.96	29.65	0.400

AFP: α-fetoprotein

NLR: Neutrophil to lymphocyte ratio

ALT: Alanine Aminotransferase

PLT: Platelet

### Comparison of therapeutic outcomes between two groups

#### Overall Survival(OS) analysis

**Figure1:**
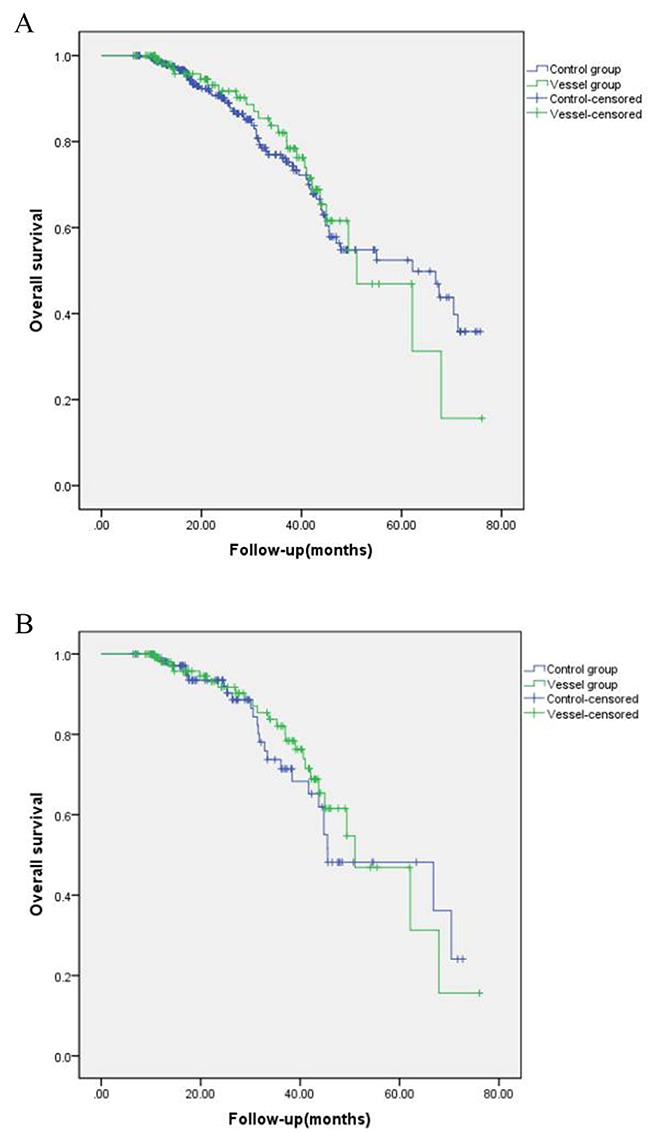

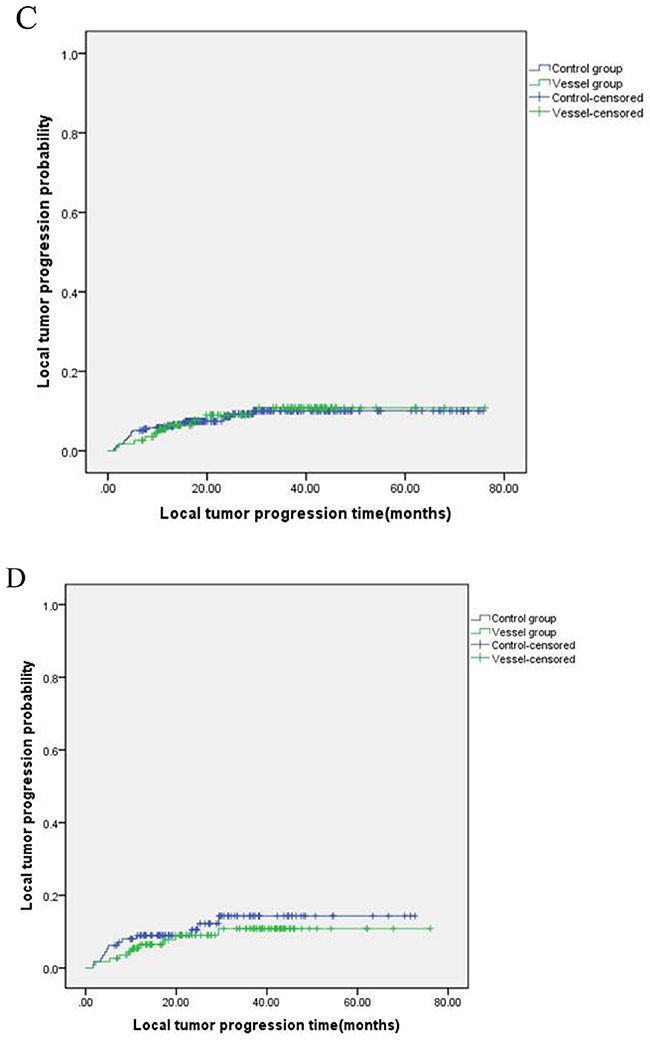
Kaplan–Meier curves of HCC patients with different tumor location **(A)** overall survival before matching; **(B)** overall survival after matching. **(C)** local tumor progression before matching; **(D)** local tumor progression after matching.

The estimated OS rates at 1, 3 and 5 years were 98.6%, 77.0%, and 52.5% for the 293 patients in the safe group and 98.0%, 82.0%, and 46.9% for the 113 patients in the vessel group (Figure 1A). After matching, respective 1, 3 and 5 years OS rates were 98.1%, 73.7% and 48.2% in the safe group and 98.0%, 82.0% and 46.9% in the vessel group (Figure 1B). There were no significant differences between the two groups either before or after matching (p=0.93 and p=0.62, respectively). HRs for OS were not significantly different between groups, regardless of matching (HR = 0.98 [95% CI: 0.62, 1.56], P = 0.92 for univariate analysis of overall data; and HR = 0.87 [95% CI: 0.50, 1.51], P = 0.62 for matched data). Multivariate analysis conducted by adjusting for propensity score in the overall data also did not show a significant difference in terms of OS between the two groups (HR = 1.05; 95% CI: 0.48, 2.30; P = 0.90).

#### LTP analysis

The cumulative 1, 3 and 5 year LTP rates were 6.2%, 10.1% and 10.1% in the safe group and 6.5%, 10.8% and 10.8% in the vessel group, respectively (p = 0.78, Figure 1C). After matching, the cumulative 1, 3 and 5 year LTP rates were 9.0%, 14.3% and 14.3% in the safe group and 6.5%, 10.8% and 10.8% in the vessel group, respectively (p = 0.47, Figure 1D). Multivariate analysis conducted by adjusting the propensity score in the overall data also showed no significant difference between the two groups (HR = 0.65; 95% CI: 0.26, 1.61; P = 0.35).

### Analysis of risk factors for therapeutic outcomes

In univariate analysis, tumor size and total ablation time during MW ablation were potential risk factors for LTP (P< 0.1). In multivariate analysis, tumor size (HR = 1.4; 95% CI: 1.08, 1.91; P = 0.003) was independent risk factors for LTP. High α-fetoprotein (AFP) level, neutrophil to lymphocyte ratio (NLR), platelet (PLT) and highest ablation power were significant risk factors for OS in univariate analysis. However, high AFP level (HR = 1.88; 95% CI: 1.19, 2.97; P = 0.007), NLR (HR = 1.17; 95% CI: 1.07, 1.27; P < 0.001, and PLT (HR = 0.90; 95% CI: 0.83, 0.98; P = 0.02) were associated with OS in multivariate analysis (Table 2).

**Table 2:** Univariate and multivariate analyses of LTP and OS

	LTP				OS			
	univariate analysis		multivariate analysis		univariate analysis		multivariate analysis	
**Factors**	HR	P	HR	P	HR	P	HR	P
**Age**	0.99	0.88			1.01	0.56		
**NO. of men**	0.71	0.38			1.23	0.57		
**Tumor size**	1.44	0.01	1.44	0.03	1.14	0.24		
**AFP level >200 ng/mL**	0.88	0.78			2.08	0.01	1.88	<0.00
**Origin of chronic liver disease**	1.33	0.13			1.16	0.43		
** HBV**								
** HCV**								
** Others**								
** None**								
**Presence of liver cirrhosis**	0.81	0.76			2.52	0.36		
** yes**								
** no**								
**Child-Pugh class**	0.59	0.67			1.36	0.63		
** Class A**								
** Class B**								
**NLR before ablation**	1.05	0.50			1.14	0.03	1.17	<0.00
**ALT**	0.99	0.38			1.01	0.86		
**PLT**	0.99	0.43			0.88	0.004	0.90	0.01
**Ablation power (W)**	0.95	0.27			0.51	0.003		
**Abaltion time(s)**	1.00	0.07			1.00	0.75		
**Adjacent to large vessels**	1.66	0.14			0.87	0.62		

HBV: Hepatitis B virus

HCV: Hepatitis C virus

### Complications

The complication rate associated with MW ablation was 3.2% (13 of 406 patients) in the overall data. No patients in either group had immediate major complications associated with injury to the perihepatic structures. In the overall data, there was a higher incidence of complications in the vessel group than in the safe groups (6.19% vs 2.04 %, P <0.05). But for the matched data, no significant differences were found between two groups (4.42% vs 7.10%, P =0.72) (Table 3). Besides, in the vessel group, one patient was found thrombosis in the right portal vein and the central part of the left portal vein the second month after ablation, which disappeared 3 months later without any management.

**Table 3:** Complications after MW ablation

Complications	All data			Matched data		
	Safe group	Vessel group	P	Safe group	Vessel group	P
**Major**						
Pleural effusion	3	2		2	2	
Tumor seeding	1	2		1	2	
Abcess	1	0		1	1	
**Minor**						
Thrombosis	0	1		0	1	
Hemorrhage	1	2		1	2	
**Total**	**6**	**7**	**0.03**	**5**	**8**	**0.39**

## DISCUSSION

Investigators in previous studies suggested that the vicinity of large vessels was one of possible risk factors for LTP after RF ablation [4, 17]. Despite the susceptibility to the heat sink effect, the weaker active heating ability of RF was thought to be another reason for the LTP after ablation of tumors adjacent to large vessels [10]. Kang et al. found that after using multiple or large electrodes and a more powerful generator, long-term therapeutic outcomes of RF ablation as first-line treatment for small perivascular HCC (3 cm or smaller) were similar to those for nonperivascular HCC [14]. Compared with RF, MW had advantages of higher thermal efficiency and less susceptibility to the heat sink effect [12], which could help to enhance the effectiveness of MW ablation for tumors adjacent to large tumors. However, clinical studies about the effectiveness of MW ablation for HCC adjacent to large vessels were very limited. Huang et al. reported the long-term results of MW ablation for liver tumors adjacent to large vessels and they found that the vicinity of large vessels itself did not significantly influence local tumor control and OS after MW ablation for HCC [15]. Yu et al. also found that the LTP rate was not increased by the position in relation to the vessels after MW ablation for malignant liver tumors [18]. Conclusions of both studies corresponded well to our study. However, the inclusion criteria of both studies were broad. The included recurrent HCC in Huang et al.'s study might suppress the survival results, and previous treatment before ablation could influence the efficacy observation of MW ablation itself. The inclusion of liver metastases in Yu et al.'s study might not indicate the right conclusions because tumor pathology was believed as a prognosis risk factor after ablation. In most retrospective studies, researchers did not balance the clinical characteristics of patients between groups, which could have resulted in incorrect or contradictory conclusions. To our knowledge, our study was the first to use PSM analysis to illustrate the value of MW ablation as curative therapy for early-stage HCC adjacent to large vessels.

We found no significant differences in the vessel and safe groups for long-term therapeutic outcomes, including LTP and OS for MW ablation used as a first-line treatment for the early-stage HCC. For major complication rates, the vessel group had a higher rate than the safe group in the overall data, while no significant difference was seen between the groups after matching. We proposed that the intergroup differences in major complication rates before matching were caused by different baseline characteristics between groups. Patients with larger tumors and higher AFP level were more often assigned to the vessel group in the overall data. These disproportions could influence the outcome of tumor control. Previous studies have concluded that larger tumor size and higher AFP level were associated with LTP and tumor recurrence [19–22]. So direct comparations of baseline and clinical data in two groups were not proper and could put lots of bias to the results.

A possible explanation for no significant differences in OS between groups before or after PSM was thermal ablation as a first-line therapy only accounted for a small part of treatment history for HCC. The HCC recurrence rate was estimated to be relatively high up to 50% at 5 years after initial treatment, owing to intrahepatic metastasis or de novo carcinogenesis from the remaining preneoplastic liver [19, 20]. The high AFP level, lower NLR and lower PLT were found to be associated with poor OS outcome in our study, which were similar with previous studies [21–24]. Tumor size was no longer a poor prognostic factor for OS, but acted as a risk factor for LTP in our study. As mentioned above, ablation was only a small part of long-term patient care for HCC and LTP was not a risk factor for OS [19]. In contrast, factors that influence body state, such as systemic inflammatory response and immune response, might determine the OS in long term.

Another major concern of MW ablation for HCC adjacent to large vessels was the risk of complications. Due to the heat-sink effects of large vessels, higher ablation power or longer ablation time was needed to guarantee the complete coagulation of tumors including safe margins. But high energy output might injure the surrounding vessels. It might cause the occlusion of blood vessels and segmental infarction of liver [7–9]. In our study, thrombosis was found in one patient druing the regular follow-up. The patient has no syndrome and the liver function test was normal. The thrombosis finally disappeared without any treatment. We speculated that the thermal injury to the vessels was minor and recoverable. The circulating blood could take heat away and protect the vascular endothelium from severe damage during ablation. Previous studies also reported that an increase in intratumoral pressure due to thermal heating could cause dislodgement and spread of cancer cells around the ablation zone by means of blood vessels [10, 11], resulting in multiple or infiltrative tumor recurrence. The exact reason of aggressive tumor recurrence wass still unclear. Kang et al. also suggested that periportal tumor location and younger patient age were significant risk factors for aggressive recurrence after ablation for HCC [25]. But Kang et al.’ study was flawed by the inefficiency of single electrode. Modified ablative technique was recommended to decrease the risk of aggressive recurrence [26]. Hocquelet et al. recommended MW ablation as an effective way to decrease heat-sink effects and achieve satisfying margins to prevent aggressive risk [26]. In our study, no multiple or infiltrative tumor recurrence were encounted. It might attribute to the high thermal efficiency of MW device and rich experience of operators. All operators in this study had more than ten years’ experience in MW ablation and had a good control of thermal field during MW ablation. No significant differences were found between groups for major complications after balancing patients’ baseline clinical characteristics, though the vessel group showed higher major complication rate than the safe group in overall data. The use of thermal monitoring also contributed to reduce the risk of major complications and enhanced thermal effect. Our study well proved that MW ablation had favorable ability in treating HCC adjacent to large vessels.

Our study had several limitations. First, this was a retrospective study lacking randomization. Therefore, inherent selection bias was unavoidable. However, this bias was limited by the similar baseline characteristics between the two groups using PSM analysis. Second, this was a single-center study, with a large volume of MW ablation. Thus, careful consideration is needed before generalizing our results to other settings. Third, although we tried to logically define the technical difficulty of MW ablation and followed the example of previous studies in defining high-risk locations of HCC, use of adjuvant techniques in high risk locations could not accurately represent the levels of technical difficulties during MW ablation.

## MATERIALS AND METHODS

### Patients

This retrospective study was approved by the institutional review board of Chinese PLA general hospital and was exempt from the requirement to obtain informed consent. Between January 2010 and April 2016, 6235 patients underwent MW ablation for hepatic tumors at our department. Of these, 406 patients (322 men, 84 women; mean age, 59 years; range, 24–95 years) with HCC lesions were included in this study according to the following inclusion and exclusion criteria: (a) a single nodule with a diameter < 5 cm or a maximum of three nodules with a diameter < 3 cm; (b) tumors in safe location or adjacent to large vessels; (c) treatment with percutaneous MW ablation with ultrasonographic guidance, (d) no prior therapy for HCC lesions before ablation, (e) Child-Pugh class A or B classification with Eastern Cooperative Oncology Group performance status of 0 (BCLC stage 0 or A), (f) absence of vascular invasion and extrahepatic metastasis on contrast enhanced images at the time of diagnosis, (g) more than 6 months of follow-up.

Large vessels were defined as vessels (portal vein, hepatic vein, inferior vena cava) of which diameters being equal or larger than 3 mm. HCC lesions adjacent to large vessels were defined as tumors located less than 5 mm from large vessels. Tumor in safe location was defined as tumor away from large vessels, diaphragm, gastrointestinal tract, gallbladder, pancreas, hepatic hilum and major bile duct. Diagnosis of HCC was confirmed according to findings of pathologic examination or the current practice guidelines of the American Association for the Study of Liver Diseases [27–28]. Technical success (TS) was defined as treatment of a tumor according to protocol and achievement of complete tumor coagulation during or shortly after the procedure. Technique efficacy (TE) was defined as no enhancement in any areas of the tumor in images obtained 1 month after MW ablation. Patients were classified into either the vessel group (n = 113) or the safe group (n = 293) by two authors in consensus who were blinded to clinical outcomes at the time of data collection.

### MW ablation procedure

All MW ablation procedures were performed by experienced doctors (P.L. and X.L.Y., each with 20 y of experience, Z.G.C. and Z.Y.H, each with 15 y of experience) in our department. Color Doppler and grayscale ultrasound (US) were performed to choose the safest needle access. Local anesthesia was first induced with 1% lidocaine and then the antenna was introduced into the target area of the tumor. In the multiple-needles procedure two or three prefixed puncture lines were made. Two or three active needle antennae directly connected to the MW generator were inserted into the tumor in parallel 1-2.5 cm apart. Typically, antenna was usually deployed parallel to vessels to decrease damage to large vessels in tumors adjacent to large vessels. After placing all the planned antennae (breathing cooperation required from the patient was needed to complete the insertion), venous conscious analgesia-sedation was induced with propofol (Diprivan; Zeneca Pharmaceuticals, Wilmington, Del) and ketamine (Shuanghe Pharmaceuticals, Beijing, China) associated with standard hemodynamic monitor monitoring. At each insertion, the tip of the needle was placed in the deepest, most remote, portion of the nodule under US guidance to enable the tip to be easily monitored in the absence of any disturbance caused by microbubbles during ablation. If necessary, due to tumor size, multiple overlapping ablations were usually needed to envelope the entire tumor with a least 5mm margin. In general, the microwave energy application was set at 50-80 W for 5-10 min in a session. The region of ablation was monitored by real-time US monitoring. MWA emission was stopped when the hyperechoic zone covered the entire tumor including a safety margin.

Thermal monitoring was used in some cases to ensure favorable effects and few complications. In the vessel group, a 20-gauge thermocouple connected to the MW device was inserted next to the surrounding large vessel of tumor under US guidance in 31 cases. If the temperature measured by the thermocouple reached 54°C, MW emission was stopped immediately and was restarted when the temperature became lower than 45°C [29]. This condition would continue until the entire tumor was completely covered by the hyperechoic micro-bubbles under gray-scale US. Thermal monitoring was also used in 8 cases in the safe group. The thermocouple was inserted 5-10 mm outside the tumor. If the measured temperature did not reach 60 °C by the end of treatment and did not remain at 54 °C for at least 3 min, the treatment was prolonged until the desired temperature was reached [12]. Overheating could also be avoided by thermal monitoring, thus decreasing the incidence of complications. For some small tumors that were difficult to visualize on US, CEUS was used to detect the tumor and guide the placement of antenna.

### Follow-up

Contrast-enhanced imaging (CE-MRI, CE-CT or CEUS) were performed within 3 days after the ablation to assess TS. Once residual tumor was recognized, additional ablation would be performed to achieve complete coagulation. CE-MRI, CE-CT or CEUS was used to monitor local recurrence or hepatic metastasis in 1 mo, every 3–4 mo in the first year after ablation and then every 6 mo the following years. LTP was defined as enhancing foci reappeared in the ablation zone or less than 2.0 cm from the margins. Recurrent tumors identified during follow-up were treated with optimal second-line treatment such as thermal ablation, transcatheter arterial chemoembolization, surgical resection, radiation therapy, sorafenib, and liver transplantation according to the recommendations of a multidisciplinary tumor board, based on liver function, patient performance, and recurrent tumor characteristics [14, 19]. Therapeutic outcome was assessed by LTP, OS and complications. The observation time for survival analysis was defined as the interval between the first MW ablation and either the incidence of the event or the last visit to our outpatient clinic before April 30, 2016.

### Statistical analysis

For the overall data, comparison of baseline and clinical variables between vessel group and safe group was assessed by using the t test or the Mann-Whitney U test according to normality for continuous variables (age, tumor size, NLR, ALT, PLT and follow-up time) and by using the x^<sup>2</sup>^ test for categorical variables (gender, AFP level, origin of chronic liver disease, cirrhosis and Child-Pugh class). Cumulative incidence rates for LTP and OS rates were estimated by using the Kaplan-Meier method and differences between groups were compared by using the log-rank test. Analysis of LTP and OS involved the use of a Cox proportional hazards model to assess the association of possible risk factors in univariate and multivariate analysis.

**Figure 2:**
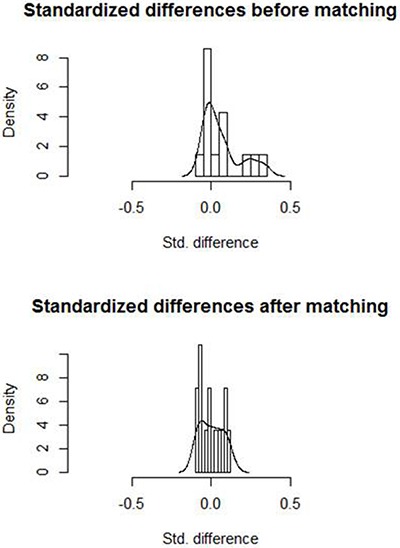
Histograms with overlaid kernel density estimates of standardized differences before and after matching Standardized differences were centralized around zero, indicating a good matching.

To reduce the effect of selection bias and confounding [Figure 2], we estimated propensity score by means of logistic regression and performed 1:1 patient matching on the basis of each patient's propensity score [30]. Variables included in the propensity score model were age, sex, tumor size, AFP level, origin of chronic liver disease, liver cirrhosis, Child-Pugh class, NLR before ablation, ALT and PLT. Comparison of groups in matched data were executed by means of the paired t test or the Wilcoxon signed rank test for continuous variables and the McNemar test for categorical variables.

Prognostic factors for OS and LTP were assessed by using Cox proportional hazard models in univariate and multivariate analyses for all patients, not just the propensity score matched patients. Potential prognostic factors included all baseline covariates used in PSM, ablation power, time and treatment modality. Major complication rates were also compared between the two groups by using all data and matched data with the McNemar test. Statistical analyses were performed by using SPSS version 22.0 software for windows statistical package, the R X642.15.1and REssentials 22.0. A P value less than 0.05 was considered to indicate a statistically significant difference.

## CONCLUSION

In summary, the differences in LTP, OS, and major complication rates of percutaneous MW ablation for early-stage HCC as a first-line treatment were not significant between the vessel group and safe group. Additionally, tumor location adjacent to large vessels did not have an adverse influence on LTP and OS independently. MW ablation is an effective and safe way to treat early-stage HCC adjacent large vessels.
